# Application of different fertilizers on morphological traits of dill (*Anethum graveolens* L.)

**DOI:** 10.1186/s13588-014-0004-z

**Published:** 2014-06-27

**Authors:** Fatemeh Nejatzadeh-Barandozi, Fathollah Gholami-Borujeni

**Affiliations:** 1 Department of Horticulture, Faculty of Agriculture, Islamic Azad University, Khoy Branch, Khoy, Iran; 2 Social Determinants of Health Research Center and Environmental Health Engineering, School of Health, Urmia University of Medical Science, Urmia, Iran

**Keywords:** Anethum graveolens, Nitroxin, Biofertilizer, Chemical fertilizer, Essential oil

## Abstract

**Background:**

The aim of this study was to evaluate the effects of nitroxin biofertilizer and chemical fertilizer on the growth, yield, and essential oil composition of dill. The experiment was conducted under field condition in randomized complete block design with three replications and two factors.

**Results:**

The first factor was the concentrations of nitroxin biofertilizer (0%, 50%, and 100%) of the recommended amount (1 l of biological fertilizer for 30 kg of seed). The second factor was the following chemical fertilizer treatments: no fertilizer (control) and 50 and 100 kg ha^−1^ urea along with 300 kg ha^−1^ ammonium phosphate. Different characteristics such as plant height, number of umbel per plant, number of umbellet per umbel, number of grain per umbellet, 1,000 seed weight, grain yield, biological yield, and oil percentage were recorded. According to the results, the highest height, biological yield, and grain yield components (except harvest index) were obtained on biological fertilizer. The results showed the highest essential oil content detected in biological fertilizer and chemical fertilizer. Identification of essential oil composition showed that the content of carvone increased with the application of biofertilizers and chemical fertilizers. The results indicated that the application of biofertilizers enhanced yield and other plant criteria in this plant.

**Conclusions:**

Generally, it seems that the use of biofertilizers or combinations of biofertilizer and chemical fertilizer could improve dill performance in addition to reduction of environmental pollution.

## Background

Plant nutrients are essential for the production of crops and healthy food for the world's expanding population. The use of chemical fertilizer, organic fertilizer, or biofertilizer has its advantages and disadvantages in the context of nutrient supply, crop growth, and environmental quality. The advantages need to be integrated in order to make optimum use of each type of fertilizer and achieve balanced nutrient management for crop growth [[1]].

Biological fertilizers are complex of some microorganisms that mobilize main nutrients from unavailable form to available form and can improve root system and seed germination. Presently, these fertilizers are considered as a replacement for chemical fertilizers to improve soil fertility and crop production in sustainable agriculture which is based on ecological principles [[2]]. Regarding the importance of medicinal plants and their role in human health, it is imperative to increase their biomass without the application of harmful chemical fertilizers, pesticides, and herbicides. The most important advantages of growth-promoting bacteria include production of growth-inducing and growth-regulating hormones, development of root system, improving water and nutrient uptake [[3]], improving seed germination and generation of plantlets [[4]], interaction effect with rhizobiums, improving availability of phosphorous to the plants, biological fixation of nitrogen [[5]], generation of ionophores especially siderophores, and production of some antibiotic compounds such as bacteriocins to control infections [[4]]. The application of biofertilizers *Azospirillum* and *Azotobacter* in the medicinal plant *Salvia officinalis* was reported to increase plant height and shoot dry and wet weights [[6]]. Also, a study by Ratti et al. [[7]] showed that simultaneous application of mycorrhiza fungus with *Azospirillum* and *Bacillus* increased biomass in the medicinal plant *Cymbopogon maritinii*. Moreover, in *Thymus vulgaris*, the application of biological fertilizers made a significant increase in the plant growth [[8]].

Dill (*Anethum graveolens* L.) is an aromatic annual grassy plant belonging to the Umbelliferae family that originally comes from Eastern Mediterranean. The entire vegetative organ contains essence. The most important essential oil compounds in this plant are d-carrone and phellandrene, and the most important compounds from the fully grown seeds are d-carrone and limenene. Combined application of organic fertilizer and urea fertilizer or the combination of urea fertilizer and polyamines significantly increases yield, vegetative growth, and evaluations on the chlorophyll index [[9]]. The aim of the present study was to investigate the effects of biofertilizers and chemical fertilizers on the growth characteristics and yield of *A. graveolens* L. and, consequently, reducing the application of chemical fertilizers.

## Methods

This experiment was conducted at an Agriculture Research farm in Tabriz (46° 39′ E, 38° 36′ N) during 2012. The soil texture was sandy clay and results of the soil analysis are shown in Table 1. The first factor tested three levels of nitroxin biofertilizer 0%, 50%, and 100% of the recommended amount (1 l of biofertilizer for 30 kg of seed). The second factor tested the following levels of chemical fertilizer: no fertilizer (control) and 50 and 100 kg ha^−1^ urea recommended along with 300 kg ha^−1^ ammonium phosphates. The field was prepared by cultivating and twice perpendicular disc harrowing followed by smoothing with leveler and then making furrows in March. Locally available seeds of dill were then sown in 4 × 2 plots of six rows in mid-April. Potassium fertilizer was applied to the land evenly into the soil base at 100 kg of potassium sulfate per hectare. Nitrogen was distributed evenly in the respective plots as follows: one third at the 5 to 4 leaf stage and another third at the emergence of the inflorescence emergence and then pre-respective irrigated plots were broadcast between rows. Seeds inoculated with nitroxin biofertilizer were stored in a black plastic in a cool place until planting time. The maximum time between inoculation of seeds and planting was about 5 h, and irrigated cultivation took place 1 week after emergence on 20 June. The sowing was done in 4 × 2 plots with six rows in each plot. The rows were set 30 cm apart from each other, and thinning was done when plants were in 4 leaf stage so that in each row, the plants were 20 cm apart. During the experiment, no herbicides, pesticides, or fungicides were applied. In order to supply the plants with nutrients, recommended amount of manure (rotten manure, 10 tons per hectare) and phosphate fertilizers prepared in triple phosphate (50 kg per hectare) were applied to the field. Weeding was done at two stages, 20 and 45 days after sowing. Flooding irrigation was done every 7 days.

**Table 1:** **Soil chemical characteristics of the experimental area at 0- to 20-cm depth (Tabriz, 2012/2013)**

pHCaCl2	P (mg dm^−3^)	O.M. (%)	Ca (cmolc dm^−3^)	K	N (%)	Ec (ds/m)	C.C.C.	***V***(%)
7.77	9.52	1.14	1.40	0.46	18	2.85	7.02	33.21

O.M., organic matter; C.C.C., cationic change capacity; Ec, electrical conductivity; *V*, basis saturation.

In order to measure the dry weight of the plants, samples were harvested and put into numbered paper bags to be sent to the laboratory. Samples were oven-dried at 50°C for 72 h and then weighed with a digital scale with ±0.01 g error of measurement. In order to observe yield compounds before harvesting, the marginal rows in each plot and also plants grown up to 50 cm from the ends of each row were set aside, and ten plants were randomly selected from each plot. The number of seeds in each spike, number of spikes per plant, number of spike in each florescence, dry and wet weights of the plants, seed performance in each plant, and weight of 1,000 seeds were recorded. Finally, statistical analysis was carried out using SPSS software and the relevant graphs were prepared using Excel. Means were compared using Duncan test (*p* ≤ 0.05).

## Results

### Plant height

The findings are displayed in Table 2. Comparison of the means for various treatments suggests that nitroxin and nitroxin × nitrogen resulted in significant effect in the plant height, and the highest plants were observed in the samples treated with nitroxin biofertilizers (Table 3).

**Table 2:** **Duncan's multiple range tests analysis for evaluated traits**

Treatments	Height of plant	Biological yield	Seed yield	Number of umbel per plant	Number of umbellet per umbel	Number of grain per umbellet	Thousand seed weight	Harvest index	Essential oil percentage	Essential oil yield
Control	55.00 c	3,033 c	1,005 c	10.67 c	7.00 c	13.00 a	3.02 c	33.10 b	2.96 c	29.74 c
Nitroxin	79.67 a	3,475 a	1,320 a	20.03 a	13.03 a	16.00 a	3.00 a	36.00 a	3.96 a	52.27 a
Nitrogen	70.09 b	3,215 b	1,140 b	14.30 b	10.11 b	14.00 a	3.09 b	35.02 ab	3.59 b	40.92 b
Nitroxin × nitrogen	77.43 a	33.70 a	1,270 a	17.30 a	10.56 b	15.00 a	3.09 b	37.01 a	4.01 a	50.92 a

Different letters indicate statistically significant differences.

**Table 3:** **Variance analysis of nitrogen and nitroxin on evaluated traits of (** ***Anethum graveolens*** **L.)**

	***df***	Height of plant	Biological yield	Seed yield	Number of umbel per plant	Number of umbellet per umbel	Number of grain per umbellet	Thousand seed weight	Harvest index	Oil percentage	Oil yield
Block	2	0.037	403.862	383.861**	0.106	0.005	0.26	0.0005	0.446*	0.004	3,649.354
Nitroxin	3	68.826**	91,422.843**	11,110.33**	191.16**	10.061**	91.378**	0.001**	0.99	1.77**	1,525,732.09**
Nitrogen	2	3.919**	2,381.861**	481.361**	0.679	0.880**	1.48**	0.0000076	0.256	0.121**	96,287.921**
Nitroxin × nitrogen	6	1.489*	394.231	63.694	0.286	0.098**	0.875**	0.00033	0.059	0.014	8,716.477
Error	22	0.411	300.376	63.740	0.209	0.014	0.029	0.00018	0.087	0.009	5,306.168
CV%		0.78	0.81	1.10	1.15	0.80	0.85	7.35	0.87	3.75	3.89

*Significant at 5% level of probability; **significant at 1% level of probability.

### Biological yield

Comparison of the means of different treatments showed nitroxin and nitroxin × nitrogen resulted in significant effect in the biological yield of *A. graveolens* L. The highest and lowest biological yields were obtained with nitroxin and control plants, respectively (Table 2).

### Seed yield

Comparison of the means of different treatments showed nitroxin and nitroxin × nitrogen resulted in significant effect in the seed yield of *A. graveolens* L. The highest and lowest seed yields were obtained with nitroxin and control plants, respectively (Table 2).

### Number of umbel per plant and number of umbellet per umbel

Analysis of variance showed that nitroxin had significant effect on the number of umbel per plant and number of umbellet per umbel (Table 2).

### Number of grain per umbellet

There is no significant effect between treatments in this trait (Table 2).

### 1,000 seed weight

The results of analysis of variance showed that the trait of seed weight under treatments of biofertilizer, chemicals, and their interactions was very significant (Table 2). The results showed that evaluations for seed weight increased with increasing concentrations of biological and chemical fertilizers. The highest evaluation for seed weight was achieved from the application of nitroxin biofertilizer (Table 2). The results of treatments comparing organic fertilizer and chemical fertilizer showed that the highest evaluation for weight of seeds was obtained by integrating biofertilizer treatment.

### Harvest index

Analysis of variance showed that the effect of combined chemical fertilizer and biofertilizer and nitroxin had significant effect on the harvest index (Table 2).

### Essential oil percentage and essential oil yield

The results showed the highest essential oil content detected in nitroxin fertilizer and nitroxin × nitrogen fertilizers. Identification of essential oil composition showed that the content of carvone increased with the application of biofertilizers and chemical fertilizers. The results indicated that the application of biofertilizers enhanced yield and other plant criteria in this plant (Table 4).

**Table 4:** **Oil composition of dill seeds affected by different fertilizers**

Compound (%)	Nitroxin × nitrogen	Nitrogen	Nitroxin	Control
Carvone	56.34 a	54.00 b	56.00 a	54.31 b
Limonene	26.10 c	28.00 b	31.30 a	31.04 a
α-Phellandrene	3.31 b	3.01 b	4.21 a	2.22 c
Dill ether	2.00 a	1.01 b	2.02 a	1.01 b
*trans*-Dihydrocarvone	4.00 b	4.01 b	4.02 b	5.01 a

Different letters indicate statistically significant differences.

## Discussion

Favorable result for the effect of *Azospirillum* and *Azotobacter* and also phosphate-solubilizing bacteria on the medicinal plant *Majorana hortensis* was reported by [[10]]. Improvement in germination indexes such as percentage and speed of germination, viability, and also the length of roots and stems of *Ocimum sanctum* and *Withania somniferum* treated with *Azospirillum* and *Azotobacter* biofertilizers, phosphate-solubilizing bacteria, nitrogen-fixing bacteria, and a combination of these fertilizers was reported by [[11]].

Many research studies have mentioned the positive effect of microorganisms on improving the growth and performance of medicinal plants. In addition to nitrogen fixation, *Azospirillum* improves root growth through generation of stimulating compounds, and these results in an increase in water and nutrient uptake and the general performance of the plant [[12]]. The most important growth-stimulating bacteria are *Azospirillum*, *Azotobacter,* and *Pseudomonas* which, in addition to biological fixation of nitrogen and solubilization of soil phosphate, considerably affect plant growth regulators especially auxin, gibberellin, and cytokinin and hence improve the plant performance. *Azotobacter* is able to produce antifungal compounds that fight plant diseases and improve viability and germination of the plantlets and, as a result, improve the overall plant growth [[1]].

The results of the present study are in agreement with [[13]] who reported that the application of biological fertilizers in *Calendula officinallis* L. and *Matricaria recutita* L. improved the performance of the shoots in these medicinal plants. Also, there is an increase in plant height and dry and wet weights of the shoots in the first and second harvest [[8],[14]].

## Conclusions

The findings of the present study suggest that the application of biological fertilizers had promising effects on dill, and this is in agreement with infrequent research studies on the effect of these fertilizers on medicinal plants. Therefore, it is recommended that the mineral nitrogen and phosphate fertilizers be replaced with biofertilizers to reduce production costs and stop damages to the environment due to the use of chemical fertilizers especially nitrogen as nitrate. The application of biofertilizer combined with growth hormones such as auxin, gibberellin, and cytokines promoted root growth that improved crop yields. Generally, it seems that the use of biofertilizers or combinations of biofertilizer and chemical fertilizer could improve dill performance in addition to reduction of environmental pollution.
